# ZFC3H1, a Zinc Finger Protein, Modulates IL-8 Transcription by Binding with Celastramycin A, a Potential Immune Suppressor

**DOI:** 10.1371/journal.pone.0108957

**Published:** 2014-09-30

**Authors:** Takeshi Tomita, Katsuaki Ieguchi, Fredric Coin, Yasuhiro Kato, Haruhisa Kikuchi, Yoshiteru Oshima, Shoichiro Kurata, Yoshiro Maru

**Affiliations:** 1 Department of Pharmacology, Tokyo Women's Medical University, 8-1 Kawada-cho, Shinjuku-ku, Tokyo, Japan; 2 IGBMC, Department of Functional Genomics and Cancer, CNRS/INSERM/Universite de Strasbourg, Strasbourg, France; 3 Graduate School of Pharmaceutical Sciences, Tohoku University, Sendai, Japan; Universidade Federal do Rio de Janeiro, Brazil

## Abstract

Celastramycin A, a small molecule that inhibits the production of antibacterial peptides in an *ex vivo* culture system of *Drosophila*, suppresses the TNFα-mediated induction of IL-8 in mammalian cells. To understand its molecular mechanism, we examined Celastramycin A binding proteins and investigated their biological functions. Our screening and subsequent pull-down assay revealed ZFC3H1 (also known as CCDC131 or CSRC2), an uncharacterized zinc finger protein, as a Celastramycin A binding protein. The knockdown of ZFC3H1 reduced *IL-8* expression levels in the TNFα-stimulated lung carcinoma cell line, LU99, and UV-irradiated HeLa cells. Based on reporter assay results, we concluded that ZFC3H1 participates in the transcriptional activation of *IL-8*. The findings of our UV-irradiation experiments implied that ZFC3H1 may indirectly interact with ERCC1 in an activated DNA repair complex. Thus, we designated ZFC3H1 as a mammalian target of Celastramycin A (mTOC).

## Introduction

Many attempts have been made to identify new reagents that may control innate immune reactions [1]–[4]. Although the innate immune system protects the host from continual attacks by pathogens, inflammatory responses may lead to unwanted consequences, such as rheumatoid arthritis [5], [6], diabetes [7], [8], or tumor metastasis [9]–[12], in some cases. Recent studies designated such an imbalance of inflammatory reactions as “homeostatic inflammation”, and suggested that the containment of inflammatory cytokines by neutralizing antibodies may prevent the formation of the pre-metastatic phase in the tumor metastasis process [13].

**Figure 1:**
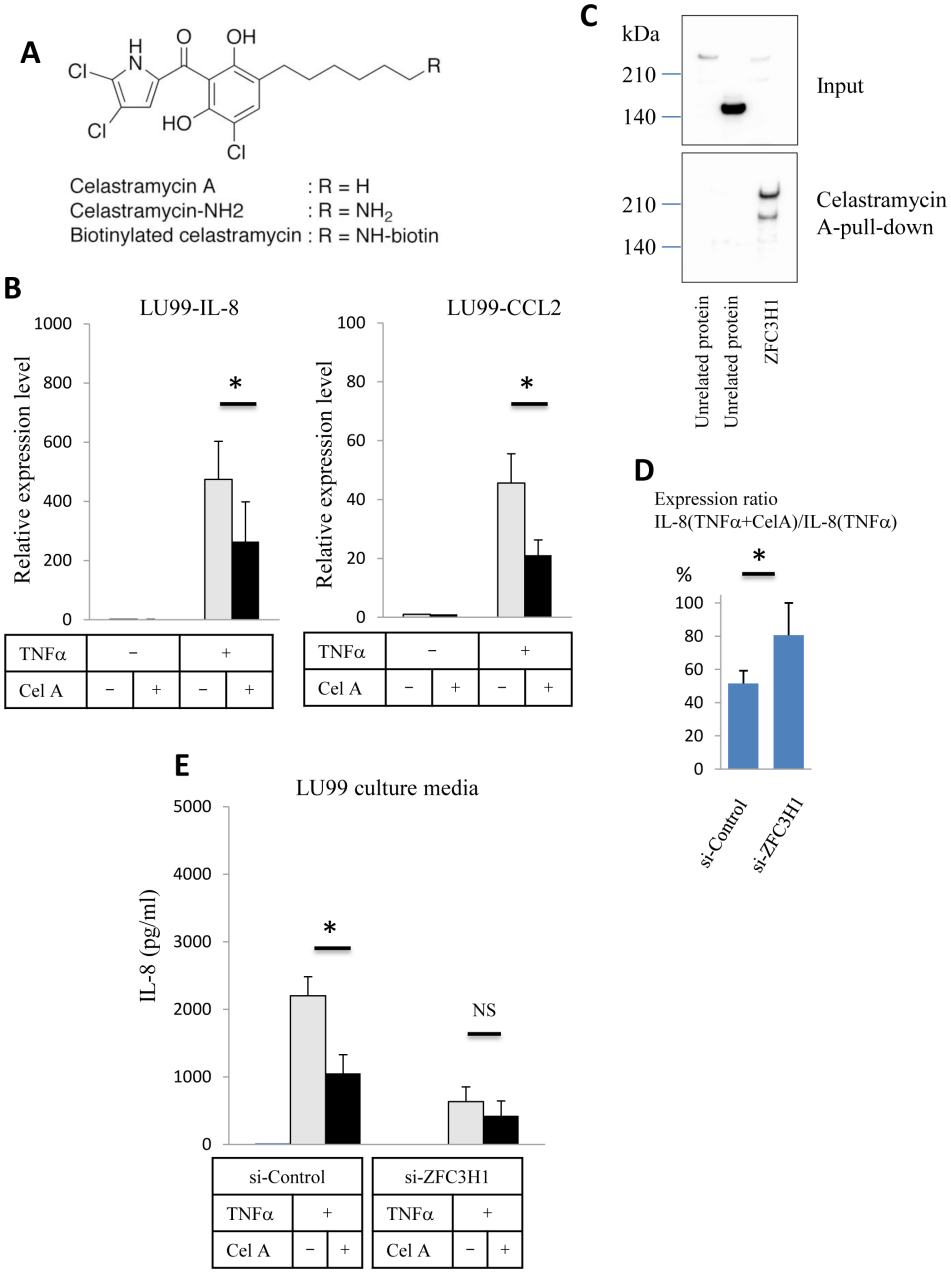
Determination of a Celastramycin A binding protein. (A) Structure of Celastramycin A (R = H) and its derivatives. The NH_2_ derivative was immobilized in magnetic beads for the pull-down assay and the biotin adduct was used for protoarray hybridization. (B) Relative *IL-8* and *CCL2* expression levels in LU99 cells determined by quantitative PCR. RNAs were isolated 90 min after the TNFα (10 ng/ml) stimulation in the absence or presence of Celastramycin A (0.1 µg/ml). Data were normalized to β-actin and control LU99 data was set as 1. Three independent experiments. (C) Celastramycin A pull-down assay. Halo-tagged ZFC3H1 or unrelated proteins with similar molecular weights (150 kDa–250 kDa) overexpressed in HEK293T cells. Input western blot data (upper panel) shows the presence of the Halo-tagged protein (anti-Halo-tag probing). The lower panel shows the Halo-tag probing western blot after Celastramycin A-immobilized bead pull-down. (D) *IL-8* expression ratio in TNFα-stimulated LU99 cells. LU99 cells were transfected with control siRNA or ZFC3H1 siRNA. RNAs were isolated 90 min after the TNFα (10 ng/ml) stimulation in the absence or presence of Celastramycin A (0.1 µg/ml). Relative *IL-8* expression ratios were calculated based on the formula to show the activity of Celastramycin A. Three independent experiments. (E) Quantitation of IL-8 in the LU99 culture media. IL-8 levels in the LU99 cell culture media were determined by ELISA. LU99 cells were transfected with control siRNA or ZFC3H1 siRNA, and culture media were harvested 6 h after the TNFα (10 ng/ml) stimulation. Celastramycin A (0.1 µg/ml) reduced IL-8 concentrations in control siRNA-transfected LU99 cells, while the effect was decreased in ZFC3H1 siRNA-transfected LU99 cells. Three independent experiments. *P<0.05. NS, not significant.

Celastramycin A, a benzoyl pyrrole-type compound (Figure 1A) originally found in a bacteria extract, arose from functional screenings using an *ex vivo* culture system of *Drosophila* [14], [15]. This compound may modulate host intracellular signaling by inhibiting the production of antibacterial peptides. Thus, its potential applications are expected to be in the inhibition of proinflammatory reactions in cells. Celastramycin A has been shown to reduce TNFα-induced IL-8 production in a mammalian cell culture assay using human umbilical vascular endothelial cells. A previous study reported that the transcription factor Relish was considered to be a likely target molecule of Celastramycin A in *Drosophila* cells; hence, NF-κB-related signal transduction molecules may be a target molecule in mammalian cells. However, detailed information on actual Celastramycin A binding molecule(s) and its molecular mechanisms has not yet been obtained. In this study, we identified the zinc finger protein, ZFC3H1, as a Celastramycin A binding protein, and determined that ZFC3H1 plays an important role in the expression of inflammatory cytokines. To the best of our knowledge, this is the first study to investigate the biological functions of ZFC3H1. The transcriptional activity responsible for the expression of *IL-8* was markedly reduced in the absence of ZFC3H1.

## Materials and Methods

### Cells and antibodies

HeLa cells that stably expressed scrambled shRNA, shERCC1, shXPA, or shXPC were kindly gifted from Dr. Denis Biard. LU99 cells were obtained from the RIKEN Bioresource Center (Tokyo, JAPAN) and maintained in RPMI1640 supplemented with 10% FBS. The ZFC3H1 (HPA007151) antibody was obtained from Sigma-Aldrich (St. Louis MO, USA). Phospho-p65 (Cat. No. 3033), IkBα (Cat. No. 4812), p38 (Cat. No. 9212), phospho-p38 (Cat. No. 9211), JNK (Cat No. 9252), phospho-JNK (Cat. No. 9251), ERK (Cat. No. 9102), phospho-ERK (Cat. No. 9101), and phospho-IkBα (Cat. No. 2859) antibodies were from Cell Signaling Technology (Danvers MA, USA). The P65 (sc-109), ERCC1 (sc-17809), lamin A/C (sc-20681) and RNA polymerase II (Pol II, sc-55492) antibodies were from Santa Cruz Biotechnology.

### Nuclear extract preparation

Cells were suspended in HEPES buffer (10 mM HEPES, pH 7.6, 15 mM KCl, 2 mM MgCl_2_, 0.1 mM EDTA, 1 mM dithiothreitol) with protease inhibitors. After brief centrifugation (1,000× g, 5 min), the cell pellet was suspended in HEPES buffer with 0.2% IGEPAL. The sample was centrifuged (1,000× g, 5 min) to separate the cytosolic fraction and nuclei, and the nuclear pellet was washed with HEPES-sucrose buffer (10 mM HEPES, pH 7.6, 250 mM sucrose, 15 mM KCl, 2 mM MgCl_2_, 0.5 M EDTA, 1 mM dithiothreitol).

### Electrophoresis Mobility Shift Assay (EMSA)

Synthetic oligonucleotides (AP-1 probe; 5′-GGAAGTGTGATGACTCAGGTTTGCCCTG-3′, and NF-κB probe; 5′-CAAATCGTGGAATTTCCTCTGACATAATGAAAAG-3′) were labeled with biotin (Biotin 3′ End DNA Labeling Kit, Thermo). Nuclear extracts were mixed with poly dI-dC (0.1 µg/ml), 1 mM dithiothreitol, and biotin labeled oligonucleotide with or without non-labeled competitor. The following shows mutant competitor sequences; mutant AP-1 5′- GGAAGTGTGATATCTGTGGTTTGCCCTG-3′, and mutant NF-κB 5′-CAAATCGTGGAATTTAAACTGACATAATGAAAAG-3′. The underlined nucleotides display mutations. Samples were electrophoresed on a 5% polyacrylamide gel using 0.5× TBE buffer. After the electrophoresis, samples were electrotransferred onto a nylon membrane for 30 min using 0.5× TBE buffer. The transferred nucleotides were fixed with UV irradiation (120 mJ/cm^2^) and biotin-labeled nucleotides were detected by using streptavidin-HRP system (LightShift Chemiluminescent EMSA Kit, Thermo).

### Chromatin Immunoprecipitation (ChIP) assay

We used ChIP-IT Express (Active Motif, Carlsbad, CA) to perform ChIP assays. Briefly, chromatin complexes isolated from cells fixed with 1% formaldehyde were sonicated (Bioruptor UCD-250, Cosmobio, Tokyo, Japan) and immunoprecipitated by control IgG or RNA polymerase II antibody. The *IL-8* promoter region contained in the immunoprecipitated samples were quantitated on a real-time PCR machine. The primers and probe set for *IL-8* promoter region was as follows; Forward primer, 5′-CATCAGTTGCAAATCGTGGA-3′, Reverse primer, 5′-AGAACTTATGCACCCTCATCTTTT-3′, probe, Roche Universal ProbeLibrary Probe #6 (Roche applied Science).

### Plasmid and siRNA

A Halo-tagged ZFC3H1 expression construct (FHC00095) was purchased from Promega (Madison WI, USA). Negative control siRNA (Cat. No. 4390843) and ZFC3H1 siRNA (Cat. No. 4392420) were obtained from Life Technologies (Carlsbad CA, USA). Regarding transfections, plasmid and/or siRNA were mixed with RNAiMAX (Life Technologies) or Hilymax (Dojindo, Kumamoto JAPAN) prior to being applied to cells.

### Celastramycin A and its derivatives

Celastramycin A was synthesized as previously reported [15]. Celastramycin A-NH_2_ and biotinylated Celastramycin A (Figure 1A) were synthesized in a similar manner to that of Celastramycin A. The details of the synthetic methods for these derivatives are described elsewhere. To make a stock solution, Celastramycin A was dissolved in DMSO to give a final concentration of 20 mg/ml, and the stock solution was then diluted in PBS prior to use.

### Protein microarray

A protoarray, a human protein microarray (PAH0525011), was purchased from Life Technologies. This was probed with biotinylated Celastramycin A followed by hybridization with a streptavidin-Alexa Fluor 647 conjugate. Fluorescence was scanned with GenePix 4100A (PerkinElmer, Boston MA USA).

### Pull-down assay

Celastramycin A-NH_2_, dissolved in 1.25 M borate buffer, pH 9.5, 1% Triton X-100, was mixed with 5 mg of Dynabeads M-280 Tosylactivated (Life Technologies) at 37°C overnight. After washing, the magnetic beads were suspended in PBS with 0.1% BSA. A small aliquot of magnetic beads (0.2 mg) was mixed with the HEK293T cell lysate overexpressing the halo-tagged protein. Beads were washed with PBS-0.1% Tween20 and mixed with SDS sample loading buffer containing β-mercaptoethanol. Samples were then electrophoresed on a 5–20% gradient gel, and electrotransferred to a polyvinylidene difluoride membrane. The membrane was blocked with blocking buffer (Nacalai Tesque, Kyoto, Japan) and probed with the anti-halo-tag (Promega) and anti-Rabbit IgG-HRP conjugate (GE Healthcare). Finally, protein bands were detected using a chemiluminescence reagent (Immunostar LD, WAKO Pure Chemicals) and a CCD camera system (ImageQuant LAS 4000mini, GE Healthcare).

### Quantitative–PCR (qPCR)

Total RNA was extracted using Trizol reagent (Life Technologies). Complementary DNA was synthesized with Primescript (Takara, Tokyo, Japan) reverse transcriptase with a random primer (Takara). Quantitative PCR analysis was performed using the SYBR Green master mixture (Applied Biosystems, Carlsbad CA, USA) and StepOneplus real time PCR system (Applied Biosystems). The following primer sets were used; β-actin, 5′-GCACAGAGCCTCGCCTT-3′ and 5′-GTTGTCGACGACGAGCG-3′; IL-8, 5′-AGCACTCCTTGGCAAAACTG-3′ and 5′-CAAGAGCCAGGAAGAAACCA-3′; CCL2, 5′-GCCTCCAGCATGAAAGTCTC-3′ and 5′-AGGTGACTGGGGCATTGAT-3′.

### Enzyme-Linked ImmunoSorbent Assay (ELISA)

IL-8 and IL-6 concentrations in the culture media were quantitated by a Quantikine ELISA system (R&D Systems, Minneapolis MN, USA).

### Reporter assay

PCR was performed using human genomic DNA with a primer set (5′- GGTACCAAATTGTGGAGCTTCAGT-3′ and 5′- CTCGAGTCTCTGAAAGTTTGTGCCTTATG-3′). The PCR product containing the *IL-8* promoter region (−238–+29) was cloned into the *Kpn*I-*Xho*I site of a Firefly luciferase pGL4.11 vector (Promega). Mutations in the AP-1, C/EBP, and NF-κB elements of this construct were introduced based on a previous study [16]. All plasmids were confirmed by nucleotide sequencing.

Cells were seeded in 24-well tissue culture plates. A total of 500 ng of the pGL4.11-based reporter construct and 5 ng of pGL4.74 as an internal control (Promega) were transfected in each well. Cells were washed once with PBS the following day and lysed with passive lysis buffer (Promega). Luciferase activity was assayed using the Dual-Luciferase Reporter Assay System (Promega) and a luminometer (GLOMAX 96 microplate reader, Promega).

### Immunofluorescence Microscopy

HeLa cells were fixed with 3.7% formaldehyde and permeabilized for 10 min at room temperature in PBS-0.1% Triton X-100 for 5 min. Cells were then incubated with PBS-2% BSA for 15 min, and with primary antibodies and 4′-6 diamidino-2-phenylindole (DAPI)-containing buffer. After washing with PBS, secondary antibodies with fluorescent dye (Alexa Fluor 488, Alexa Fluor 546, or Alexa Fluor 647) were applied. Images were obtained using a confocal laser scanning microscope (LSM710, Carl Zeiss) and processed by ZEN 2011 Image software.

### Statistical analysis

All data were expressed as the mean ± standard deviation and analyzed using the Student's t-test. P values less than 0.05 were considered significant (*). NS = not significant.

## Results

The expression of proinflammatory cytokines was markedly and rapidly increased by the TNFα stimulation. Figure 1B shows the relative *IL-8* expression levels determined by qPCR analysis of the LU99 cell line, which was derived from human lung carcinoma. The expression of *IL-8* was up-regulated >100-fold by the TNFα stimulation (5 ng/ml, 90 min), but was reduced by 50% in the presence of 0.1 µg/ml Celastramycin A. To evaluate cellular toxicity of Celastramycin A, cell viability assay was performed. The results shown in Figure S1 demonstrated that incubation with 1 µg/ml of Celastramycin A for 6 h reduced LU99 cell viability by 10% but no significant loss was seen in the lower Celastramycin A concentration region (0.01–0.1 µg/ml), whereas TNFα induced *IL-8* expression was inhibited. The expression of *CCL2* was also up-regulated approximately 40-fold by the TNFα stimulation, but was reduced by 50% following the addition of Celastramycin A. Almost the same effect was observed in a human breast carcinoma cell line (HCC1954 cells, data not shown).

Protein array screening was performed to identify Celastramycin A binding proteins. The array, on which recombinant human proteins were spotted, was hybridized with biotinylated Celastramycin A followed by fluorescence-labeled streptavidin probing. This assay identified several molecules as Celastramycin A binding proteins, and a pull-down assay confirmed that the ZFC3H1 protein bound to Celastramycin A. Figure 1C shows the results of a pull-down assay, which revealed that the N-terminal Halo-tagged full length of ZFC3H1 was concentrated by magnetic beads covalently binding Celastramycin A with a spacer group, while two other unrelated proteins failed to bind the ligand.

ZFC3H1 knockdown experiments demonstrated that the Celastramycin-dependent IL-8 reduction was canceled by ZFC3H1 siRNA transfection prior to the TNFα stimulation (Figure 1D). Quantification of the IL-8 protein in the culture media clearly showed that IL-8 levels were markedly decreased in ZFC3H1-silenced LU99 cells. Celastramycin A reduced the production of IL-8 by 50%, but did not in ZFC3H1-silenced LU99 cells, as shown in the graph comparing IL-8 levels between Celastramycin A(+) control siRNA-LU99/control siRNA-LU99 and Celastramycin A(+) ZFC3H1 siRNA-LU99/ZFC3H1 siRNA-LU99 (Figure 1E). In the IL-8 assay data, ZFC3H1 knockdown yielded more reductions than Celastramycin A treatment. This was attributed to the partial agonistic activity of Celastramycin A against ZFC3H1. On the other hand, IL-6 levels were attenuated by the addition of Celastramycin A even in the ZFC3H1-silenced LU99 cells (Figure S2). It should be noted that ZFC3H1 knockdown was also effective to reduce IL-6 production as well as IL-8 production (Figure S2). These data suggest that ZFC3H1 was also a key factor in regulation of IL-6 expression, but the effect of Celastramycin A was not limited on ZFC3H1. Thus, ZFC3H1 plays an important role in TNFα signal transduction in order to enhance the expression of downstream genes and Celastramycin A inhibits the function of this molecule to some extent.

**Figure 2:**
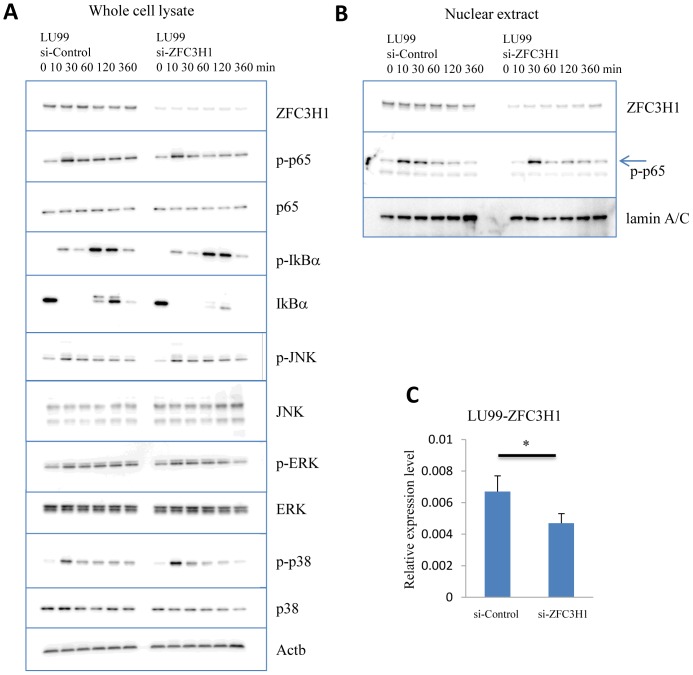
Western blotting analysis of ZFC3H1 and intracellular signaling. (A) Control and ZFC3H1 siRNA-transfected LU99 whole cell lysates were prepared 0–360 min after TNFα (5 ng/ml) stimulation. The panels show ZFC3H1, phosphor-p65, p65, phosphor-IkBα, IkBα, phospho-JNK, JNK, phosphor-ERK, ERK, phosphor-p38, p38, and β-actin blots, respectively. Three independent experiments. (B) Western blot analysis of nuclear extracts from control or ZFC3H1 siRNA-transfected LU99 cells. Cell lysates were prepared 0–360 min after the TNFα (5 ng/ml) stimulation. The panels display ZFC3H1, phospho-p65, and lamin A/C blots, respectively. In phospho-p65 western blot, phospho-p65 specific bands were arrow-marked. Three independent experiments. (C) Relative *ZFC3H1* expression levels in LU99 cells determined by quantitative PCR. Data were normalized to β-actin. *P<0.05.

Western blot analysis was used to decipher the role of ZFC3H1 in intracellular TNFα signaling. ZFC3H1 was localized in the cytosol and nucleus of LU99 cells, and no significant change was observed in the localization pattern with the TNFα stimulation (Figure 2A and B). The western blot data shows that ZFC3H1 siRNA transfection reduced the expression levels of ZFC3H1 protein by 80%, whereas RNA level quantifications pointed out modest decline of ZFC3H1 (Figure 2C). In Figure 2A, the western blots of LU99 cell total lysates with control or ZFC3H1 siRNA transfection, IkBα was phosphorylated in control siRNA-transfected LU99 cells to produce a mobility shift 5–10 min after the TNFα stimulation, and the same response was observed in ZFC3H1-silenced cells. No significant change was observed in the phosphorylation of IkBα or p65 between control and ZFC3H1 siRNA-transfected LU99 cells, suggesting that ZFC3H1 was not involved in TNFα-p65 signaling. It should be noted that minor differences in JNK, p38, and ERK phosphorylation patterns upon stimulation of TNFα between control and ZFC3H1 siRNA transfected LU99 cells were observed. Thus, Celastramycin A caused minor changes in intracellular signaling, but not enough to explain its inhibitory effect on the production of IL-8.

**Figure 3:**
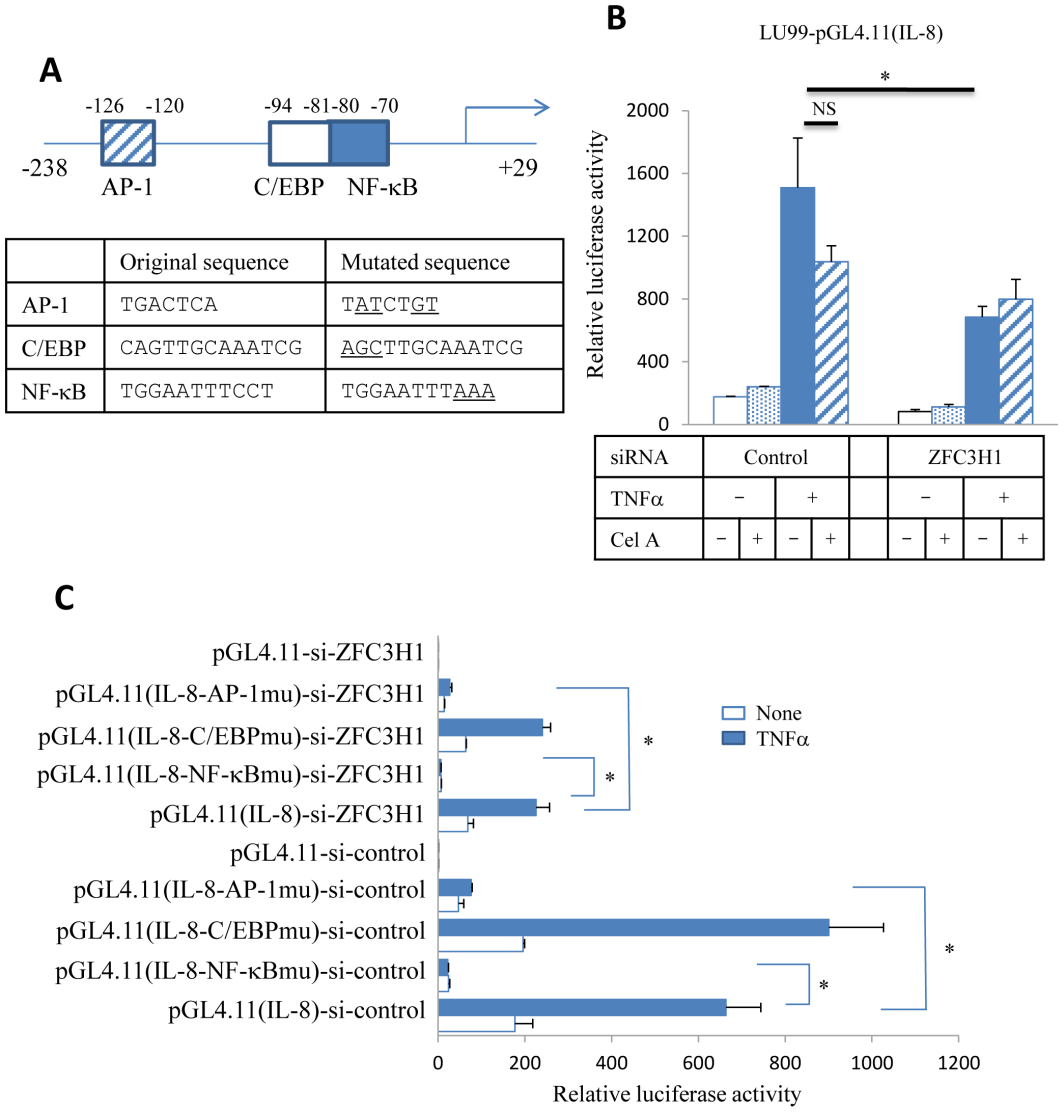
Effect of ZFC3H1 on *IL-8* gene transcription. (A) Structure of the human *IL-8* promoter. AP-1, C/EBP, and NF-κB sites are displayed. In the mutant constructs, underlined nucleotides were replaced. (B) IL-8(−238–+29)-pGL4.11 and the internal control vector, pGL4.74, were transfected into LU99 cells, and their luciferase activities were measured 6 h after the TNFα (20 ng/ml) stimulation. Firefly luciferase activity was divided by Renilla luciferase activity. Three independent experiments. (C) IL-8-pGL4.11 with or without mutations was co-transfected with pGL4.74 into LU99 cells. LU99 cells were treated with control or ZFC3H1 siRNA prior to the experiments. Cells were stimulated with TNFα (5 ng/ml) for 6 h. Firefly luciferase activity was divided by Renilla luciferase activity. pGL4.11 is a blank vector, and this data was set as 1. Three independent experiments. *P<0.05. NS, not significant.

The reporter assay very sensitively reflects the involvement of transcription factors in the gene expression study. A previous study revealed that the proximal human *IL-8* promoter region controlled *IL-8* gene expression; however, other additional elements apart from the proximal region may be able to affect transcription [17]. In the present study, we prepared a human *IL-8* proximal region containing AP-1, C/EBP, and NF-κB binding sites (Figure 3A), along with the mutant constructs listed in Figure 3A [16]–[18]. This region, cloned into the Firefly luciferase vector and transfected into LU99 cells, became a scaffold of transcription factors activated by the TNFα stimulation. The addition of Celastramycin A reduced Firefly luciferase activity, implying that Celastramycin A kept transcription factors from binding to the proximal *IL-8* promoter region (Figure 3B). Our experiments to determine the relative luciferase activity revealed that the difference between control and ZFC3H1 siRNA transfected LU99 cells was statistically significant but not between with and without Celastramycin A (Figure 3B). The latter analyses gave p-values 0.05–0.06. Celastramycin A slightly increased transcription activities, which was presented in the difference between si-control-TNFα(−) or si-ZFC3H1-TNFα(+) with and without Celastramycin A in Figure 3B. This increase was attributed to the modest effect in the reporter assay. The TNFα-mediated up-regulation of Firefly luciferase activity was observed in ZFC3H1 siRNA-transfected LU99 cells, but this was 50% less than that in control siRNA-transfected LU99 cells (Figure 3B). This accounts for the fact that IL-8 transcription was reduced by ZFC3H1 knockdown.

Site-directed mutations were introduced in the *IL-8* proximal promoter construct in attempt to narrow down the function of ZFC3H1 in *IL-8* transcription. Firefly luciferase constructs with AP-1 site mutations (IL-8-AP-1mu), C/EBP site mutations (IL-8-C/EBPmu), or NF-κB site mutations (IL-8-NF-κBmu) [17] were transfected into LU99 cells, with control or ZFC3H1 siRNA. The assay results in Figure 3C show that *IL-8* transcription was regulated by AP-1 and NF-κB in LU99 cells. Since the AP-1 or NF-κB mutation inhibited Firefly luciferase activity, this result indicates that these transcription factors could work synergistically. ZFC3H1 knockdown reduced Firefly luciferase activity while maintaining Renilla luciferase activity, which indicated that ZFC3H1 silencing had an effect on the *IL-8* promoter, but not on the thymidine kinase promoter cloned into the pGL4.74 reference vector. This result suggests that ZFC3H1 plays an important role in the AP-1-NF-κB transcription complex.

**Figure 4:**
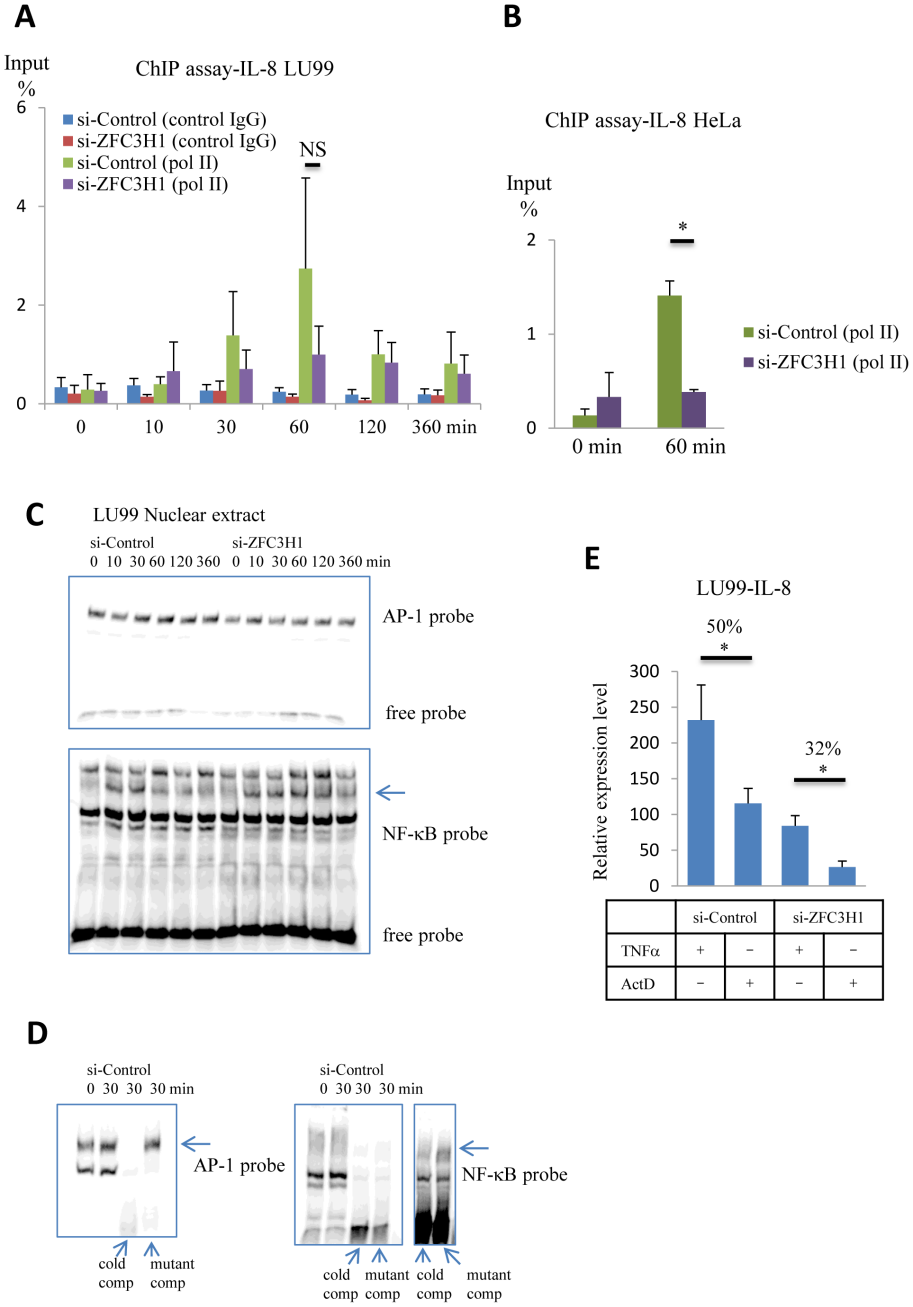
Effect of ZFC3H1 on transcription and mRNA stability. (A) ChIP monitoring the TNFα-dependent occupancy of Pol II on the *IL-8* promoter of si-control or si-ZFC3H1 transfected LU99 cell. Control IgG data are also shown. Values are expressed as percent input. Four independent experiments. (B) ChIP monitoring the TNFα-dependent occupancy of Pol II on the *IL-8* promoter of control or ZFC3H1 siRNA transfected HeLa cell. Values are expressed as percent input. Three independent experiments. (C) EMSAs were carried out using nuclear extract from control or ZFC3H1 siRNA transfected LU99 cells. AP-1 probe (upper panel) and NF-κB probe (lower panel) data are presented. In EMSA with NF-κB probe, an arrow-marked band changed its intensity upon TNFα stimulation. (D) To confirm AP-1 and NF-κB specific bindings, 200-fold excess amount of cold or mutant competitors were added in the samples. In AP-1 probe EMSA (left panel), samples prepared 30 min after TNFα stimulation were mixed with cold or mutant competitors. The left one lane shows sample prepared 0 min after TNFα stimulation as a reference. AP-1 specific binding remained unchanged in the presence of mutant competitor (marked with an arrow). On the other hand, NF-κB probe EMSA (right panel), small fraction remained unchanged in the presence of mutant competitor, which was clearly visualized in the enhanced contrast image (far right two lanes). The left one lane shows sample prepared 0 min after TNFα stimulation as a reference. (E) *IL-8* mRNA stability assay. LU99 cells were incubated with 5 ng/ml TNFα for 1 h, and then medium was switched to actinomycin D (ActD, 5 µg/ml) containing medium. The ActD samples were retrieved 2 h after the medium change. Contents of *IL-8* mRNA were quantitated by using RT-qPCR method. Data were normalized to 18S rRNA and control LU99 data was set as 1. To obtain values of TNFα (+)-ActD(−) samples as references, cells were processed immediately after the 1 h incubation with TNFα. Numbers indicate the ratio of *IL-8* mRNA remaining 2 h after the ActD treatment. Three independent experiments. *P<0.05. NS, not significant.

To investigate recruitment of basic transcription factors at the transcription start site, ChIP assay was executed. LU99 cells with si-control or si-ZFC3H1 were fixed with formaldehyde 0–360 min after TNFα stimulation were harvested and sonicated nuclear fractions were immumoprecipitated with RNA polymerase II (Pol II) antibody. The *IL-8* promoter region contained in the immunoprecipitated samples were quantitated by using our real-time PCR system and the results were summarized in Figure 4A. In both control and ZFC3H1 siRNA transfected LU99 cells-ChIP assays, Pol II yielded higher copy numbers of *IL-8* promoter region, especially 60 min after TNFα stimulation, than control IgG. Our four independent experiments retained this tendency, but we could not determine that the difference between Pol II fractions of control and ZFC3H1 siRNA transfected LU99 cells-ChIP was statistically significant. Possibly the difference were masked by the relatively large deviation due to multiple steps in the assay. Next, we carried out ChIP assay by using HeLa cells because they exhibited larger differences in the TNFα induced *IL-8* expression levels between control and ZFC3H1 siRNA transfected cells than LU99 cells. In this case a clear distinction was observed (Figure 4B).

To analyze AP-1 and NF-κB consensus sequence binding elements in the TNFα stimulated LU99 cells, their nuclear extracts were tested in EMSA. Figure 4C and D show that the AP-1 probe binding pattern appears to be constant and no critical change was observed upon TNFα stimulation. Their AP-1 specific binding was confirmed by assays with 200-fold excess amount of cold or AP-1 site mutant competitor oligo nucleotides listed in Figure 3A. In contrast to AP-1 probe, NF-κB probe gave multiple bands, and in the data a weak band appeared in a TNFα stimulation dependent manner (Figure 4C lower panel). This band became more obscure in another preparation as shown in Figure 4D right panels. This figure presents the EMSA data of NF-κB probe with 200-fold excess amount cold or NF-κB site mutant competitors described in Figure 3A. Intensity of the NF-κB specific binding signal was so weak that the real band can be recognized in the image with enhanced contrast, implying that the amount of NF-κB binding proteins in the nuclear extract was very small although NF-κB binding was absolutely necessary as shown in the reporter assay (Figure 3C).

The effect of ZFC3H1 on *IL-8* mRNA stability was also examined. Figure 4E presents relative expression levels of *IL-8* in LU99 cells. Cells were incubated with 5 ng/ml of TNFα for 1 h, and then medium was switched to 5 µg/ml actinomycin D containing medium to take 2 h incubation. Actinomycin D treatment allowed us to observe stability of mRNA by blocking mRNA synthesis [19]. Two hours after the actinomycin D treatment, amount of *IL-8* mRNA in control siRNA transfected LU99 cells, became 50% of initial value (Figure 4E, left two columns). On the other hand, in ZFC3H1 siRNA transfected LU99 cells the actinomycin D treatment reduced amount of *IL-8* mRNA by 68% (Figure 4E, right two columns). This is interpreted that *IL-8* mRNA had short life time in the absence of ZFC3H1. These results suggest that ZFC3H1 might be involved in regulation of *IL-8* mRNA stability. Addition of Celastramycin A in the actinomycin D treatment step did not change the amount *IL-8* mRNA (data not shown).

**Figure 5:**
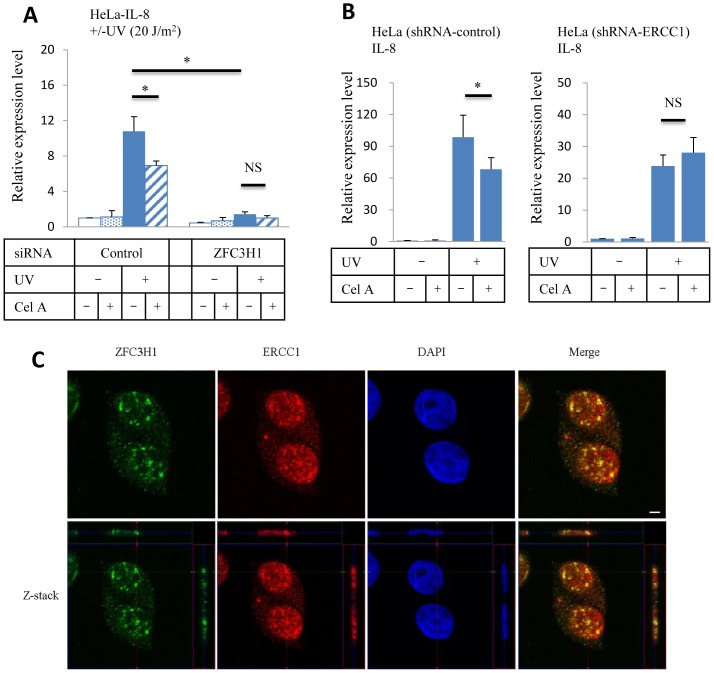
Effect of Celastramycin A on HeLa cells. (A) *IL-8* expression levels in HeLa cells transfected with control or ZFC3H1 siRNA. RNAs were isolated from HeLa cells 5 h after UV (20 J/m^2^) irradiation. The concentration of Celastramycin A was 0.05 µg/ml. Data were normalized to β-actin and control HeLa data was set as 1. Three independent experiments. (B) *IL-8* expression levels in HeLa cells with control shRNA (left) and shERCC1 (right). RNAs were isolated from HeLa cells 5 h after UV (20 J/m^2^) irradiation. The concentration of Celastramycin A was 0.05 µg/ml. Data were normalized to β-actin and control HeLa (shRNA-control, left) or HeLa (shRNA-ERCC1, right) data was set as 1. Three independent experiments. (C) Confocal microscopic images showing the localization of ZFC3H1 (green) and ERCC1 (red). Scale bar, 5 µm. *P<0.05. NS, not significant.

*IL-8* can be up-regulated by cellular stresses other than the TNFα stimulation. UV irradiation is known to indirectly enhance the expression of IL-8 in cells [20]. Figure 4 shows *IL-8* expression levels in HeLa cells following low level UV irradiation (20 J/m^2^). *IL-8* expression levels were almost the same as basal levels 1 h after UV irradiation, but were 10-fold higher after 5 h. No significant changes were observed in the expression of *CCL2* (data not shown). Incubation with Celastramycin A-containing medium following UV irradiation reduced *IL-8* expression by 20–30%, and ZFC3H1 knockdown in HeLa cells inhibited the up-regulation of *IL-8* (Figure 5A). The DNA repair complex rearrangement occurs following UV irradiation in order to fix the damaged DNA double helix. Our results suggest that ZFC3H1 may interact with a member of the DNA repair complex through basic transcription factor components [21], [22]. Several stable HeLa cell lines with shRNAs were tested to examine interactions between the DNA repair complex and Celastramycin A, and we revealed that HeLa-shERCC1 cells were insensitive to the addition of Celastramycin A to the medium (Figure 5B). Moreover, our *in vitro* experiments showed that Celastramycin A did not bind to ERCC1, and HeLa-shXPA and HeLa-shXPC cells showed the Celastramycin A-mediated inhibition of UV-induced *IL-8* expression as control-HeLa cells (data not shown). Immunofluorescence results obtained by a confocal microscope clearly displayed that ERCC1 and ZFC3H1 localized to cytosolic and nuclear regions, whereas intense fluorescence spots were observed in nuclei only (Figure 5C). A merged picture implied the co-localization of ERCC1 and ZFC3H1; however, our co-immunoprecipitation experiments using the ERCC1 or ZFC3H1 antibody did not show the complex formation of these proteins. Thus, an indirect interaction may be taking place between ZFC3H1 and ERCC1 in the activated DNA repair complex.

## Discussion

ZFC3H1, also designated as CCDC131 (coiled-coil domain containing protein), or PSRC2 (proline/serine-rich coiled-coil protein), is a large protein (1989 amino acids), holding a C3H1-type zinc finger motif in its center, and 5 tetratricopeptide (TRP) repeats and 6 half-tetratricopeptide (HAT) repeats at the C-terminal region. Although functional characterizations of this protein have not yet been reported, RNA binding and protein-protein interactions were predicted based on the presence of TRPs and HATs [23], [24]. In this respect, we have empirically demonstrated the possibility that ZFC3H1 participates in keeping *IL-8* mRNA from degradation for the first time. The reporter assays used in this study revealed that ZFC3H1 regulated *IL-8* transcription, and the HeLa cell-UV irradiation assay suggested indirect interactions between ZFC3H1 and the DNA repair complex. These interactions may occur in the C-terminal region and zinc finger motif. Our western blotting and immunofluorescence results demonstrated that ZFC3H1 was localized to the cytosol and nuclei. This localization pattern remained unaltered with the TNFα stimulation, which indicated that no apparent nuclear translocation had occurred. Moreover, ZFC3H1 knockdown caused a slight change in the TNFα-triggered intracellular phosphorylation cascade. Thus, cytosolic ZFC3H1 may have a different role from nuclear ZFC3H1.

Our previous studies showed that cytokine signals, including TNFα, TGF-β, and VEGF, derived from the primary tumor site affected lung endothelial and epithelial cells to produce a pre-metastatic phase that facilitated actual tumor cell migration. These cytokine signals, leading to the lung-specific up-regulation of S100A8 expression and subsequent enhancement in SAA3 expression, account for lung-specific tumor metastasis [10]. SAA3 functions as an endogenous TLR4/MD-2 ligand to activate downstream NF-κB signaling [11]. Thus, the S100A8-SAA3-TLR4 cascade maintains inflammatory reactions in the lung, also known as homeostatic inflammation; therefore, an anti-inflammatory reagent such as Celastramycin A is expected to mitigate inflammatory responses in the lung. Although we did not focus on the detailed molecular mechanisms of Celastramycin A in the tumor-bearing model mouse system, ZFC3H1 may be a target molecule for the prevention of tumor metastasis.

In summary, we identified ZFC3H1 as the mammalian target of Celastramycin A (mTOC). Our study has provided an insight into this protein for the first time by identifying its functions as a transcriptional regulator. The results of this study suggest that mTOC targeting therapeutics could be effective because it is involved in regulating the expression of inflammatory cytokines.

## Supporting Information

Figure S1 **Effect of Celastramycin A in LU99 cellular viability.** (A) Correlation between relative cellular viablity of LU 99 cells and Celastramycin A concentration (0–1 µg/ml) Cells were cultured with various concentrations of Cerastramycin A for 6 h. (B) relative IL-8 expression levels in LU99 cells incubated with TNFα (5 ng/ml, 90 min) in the presence of various concentrations of Celastramycin A (0–1 µg/ml). Data were normalized to β-actin and control LU99 data was set as 1. Three independent experiments yielded the similar results.
(TIF)

Figure S2 **IL-6 production in LU99 cells was attenuated by Celastramycin A.** Control and ZFC3H1 siRNA transfected LU99 cells were incubated with TNFα (5 ng/ml, 6 h) in the absence or presence of Celastramycin A (0.1 µg/ml). The culture media were assayed with an IL-6 ELISA kit (R&D Systems). *P>0.05. Three independent experiments.
(TIF)
