# Correction: ZFC3H1, a Zinc Finger Protein, Modulates IL-8 Transcription by Binding with Celastramycin A, a Potential Immune Suppressor

**DOI:** 10.1371/journal.pone.0138262

**Published:** 2015-09-11

**Authors:** Takeshi Tomita, Katsuaki Ieguchi, Frédéric Coin, Yasuhiro Kato, Haruhisa Kikuchi, Yoshiteru Oshima, Shoichiro Kurata, Yoshiro Maru

The third author’s name is incorrectly spelled. The correct name is Frédéric Coin.

## References

[1] Tomita T, Ieguchi K, Coin F, Kato Y, Kikuchi H, Oshima Y, et al (2014) ZFC3H1, a Zinc Finger Protein, Modulates IL-8 Transcription by Binding with Celastramycin A, a Potential Immune Suppressor. PLoS ONE 9(9): e108957 doi: 10.1371/journal.pone.0108957 25268596 10.1371/journal.pone.0108957 PMC4182580

