# Supplementary figures and images for: ZFC3H1, a Zinc Finger Protein, Modulates IL-8 Transcription by Binding with Celastramycin A, a Potential Immune Suppressor
Source: PLoS One. 2014 Sep 30;9(9):e108957. doi: 10.1371/journal.pone.0108957 (PMC4182580; doi:10.1371/journal.pone.0108957)

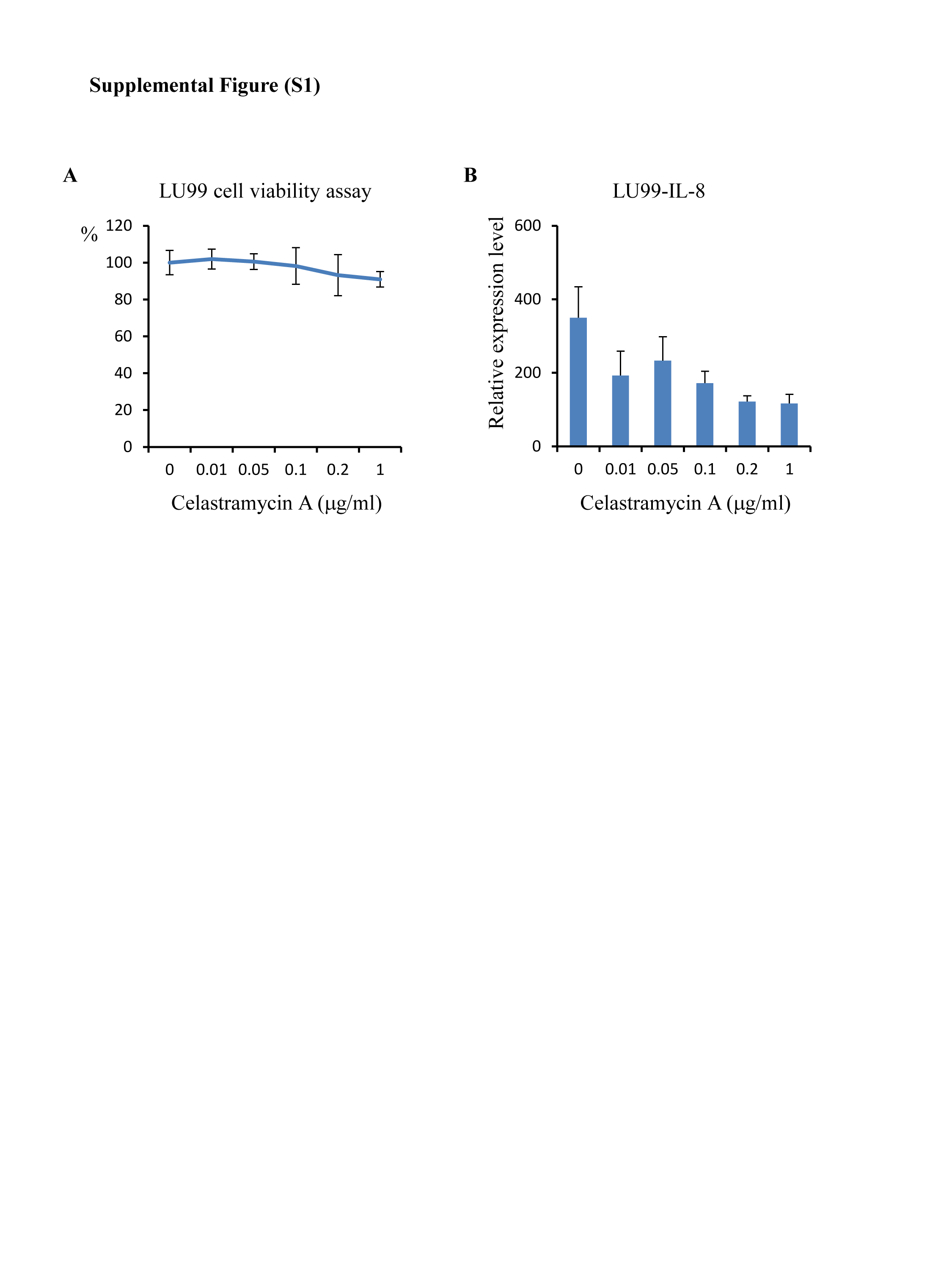

Supplement: Figure S1 — Effect of Celastramycin A in LU99 cellular viability. (A) Correlation between relative cellular viablity of LU 99 cells and Celastramycin A concentration (0–1 µg/ml) Cells were cultured with various concentrations of Cerastramycin A for 6 h. (B) relative IL-8 expression levels in LU99 cells incubated with TNFα (5 ng/ml, 90 min) in the presence of various concentrations of Celastramycin A (0–1 µg/ml). Data were normalized to β-actin and control LU99 data was set as 1. Three independent experiments yielded the similar results. (TIF) [file pone.0108957.s001.tif]

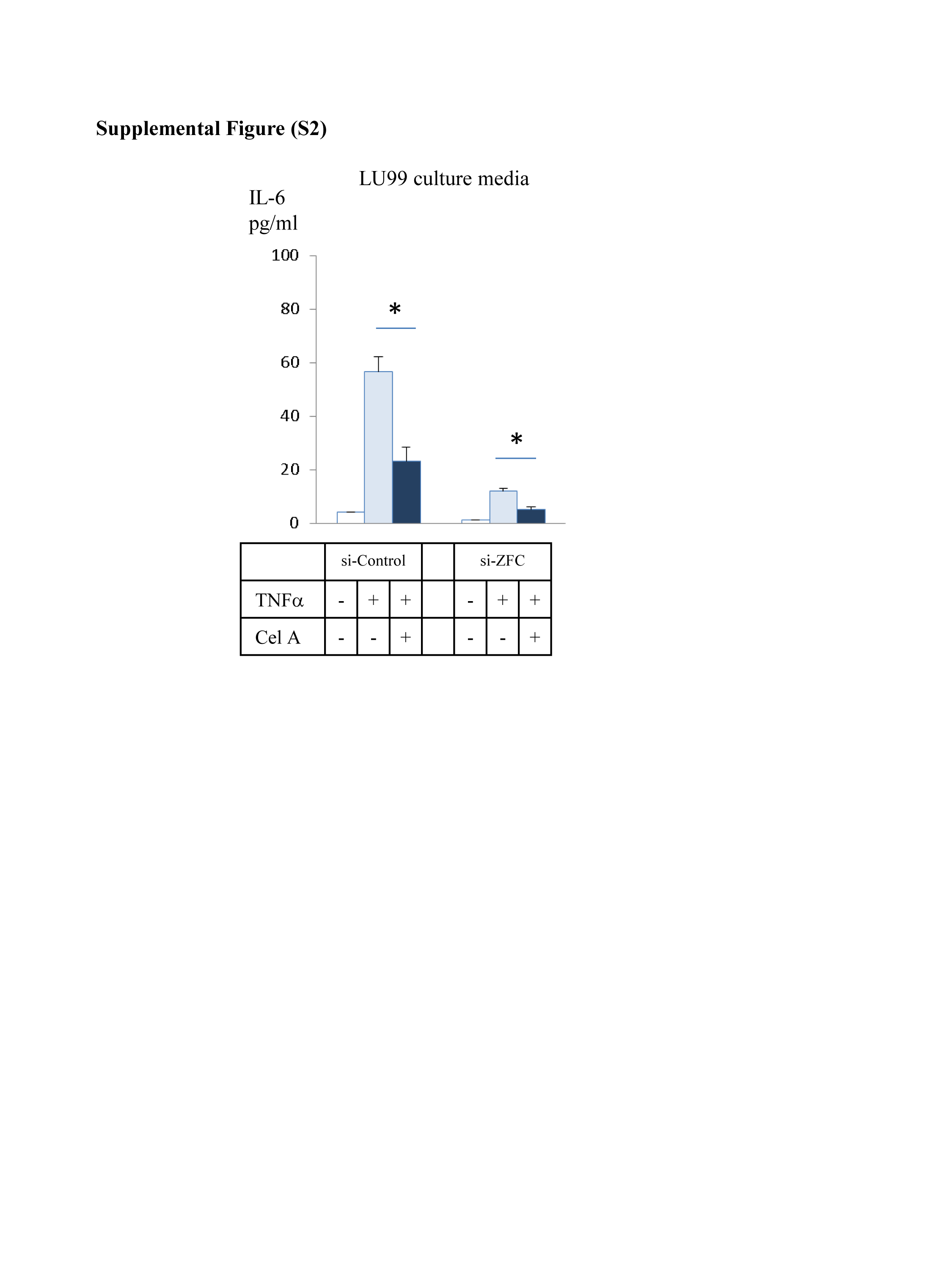

Supplement: Figure S2 — IL-6 production in LU99 cells was attenuated by Celastramycin A. Control and ZFC3H1 siRNA transfected LU99 cells were incubated with TNFα (5 ng/ml, 6 h) in the absence or presence of Celastramycin A (0.1 µg/ml). The culture media were assayed with an IL-6 ELISA kit (R&D Systems). *P>0.05. Three independent experiments. (TIF) [file pone.0108957.s002.tif]
